# A High-Temperature-Resistant and Conductive Flexible Silicone Rubber with High Phenyl Content Based on Silver-Coated Glass Fibers

**DOI:** 10.3390/polym17091187

**Published:** 2025-04-27

**Authors:** Ao Liu, Linlin Ouyang, Depeng Gong, Chaocan Zhang

**Affiliations:** School of Materials Science and Engineering, Wuhan University of Technology, Wuhan 430070, China; 331012@whut.edu.cn (A.L.); oy15070652760@163.com (L.O.); gdp@whut.edu.cn (D.G.)

**Keywords:** phenyl silicone rubber, Ag-coated glass fibers, thermal stability, conductivity

## Abstract

To enhance the high-temperature resistance of silicone rubber and meet the application requirements of flexible conductive silicone rubber under elevated temperature conditions, this study adopts a chemical modification strategy by introducing phenyl groups into the molecular chains of silicone rubber to improve its thermal resistance. High-phenyl-content hydroxyl-terminated silicone oil (MPPS) was used as the polymer backbone, and vinylmethyldimethoxysilane (VDMS) served as the chain extender. Through a silanol condensation reaction, vinylmethylphenyl polysiloxane (VMPPS) with a crosslinkable structure was synthesized, providing reactive sites for subsequent vulcanization and molding. Subsequently, needle-like silver-coated glass fiber (AGF) conductive fillers were prepared via a green and environmentally friendly electroless silver plating method. These fillers were incorporated into the phenyl polysiloxane matrix to impart electrical conductivity to the phenyl silicone rubber while synergistically enhancing its thermal resistance. Finally, thermally resistant conductive silicone rubber was fabricated through high-temperature vulcanization, and the key properties of the material were systematically characterized. The synthesized phenyl polysiloxane exhibited a number-averaged molecular weight of up to 181,136, with a PDI of 2.43. When the loading of AGF reached 25 phr, the phenyl silicone rubber composite achieved the electrical percolation threshold, exhibiting a conductivity of 7.12 S/cm. With a further increase in AGF content to 35 phr, the composite demonstrated excellent thermal stability, with a 5% weight loss temperature of 478 °C and a residual mass of 37.36% at 800 °C. Moreover, after thermal aging at 100 °C for 72 h, the conductivity degradation of the phenyl silicone rubber was significantly lower than that of commercial silicone rubber, indicating outstanding electrical stability. This study provides an effective approach for the application of flexible electronic materials under extreme thermal environments.

## 1. Introduction

With the rapid development of high-tech fields, the demand for high-performance materials under extreme service conditions continues to grow. Key components, such as high-temperature flexible sensors in electronic devices, surface temperature monitoring sensors for aircraft skins in aerospace systems [1], conductive sealing layers for automotive engine control units, and conductive sealing joints in high-temperature furnaces for industrial electrical equipment [2,3,4], require stable operation under high temperatures of 200 °C or even 300 °C. To meet the demand for a broader working temperature range in specific applications, there is an urgent need for novel high-temperature-resistant conductive materials. Common high-temperature polymer materials include silicone–fluorine rubbers and traditional organic polymers. For instance, Kareem et al. [5] prepared high-temperature polyimide/silica composites using in situ polymerization and solution blending methods, while Wu et al. [6] developed high-temperature silica/fluororubber materials. Traditional high-temperature resistant polymers such as polyimide exhibit excellent heat resistance but are difficult to process and lack sufficient flexibility, limiting their widespread use in the field of flexible electronics. Furthermore, fluorine-based materials can have a detrimental effect on the atmosphere, and prolonged use by humans may lead to fluorosis. As a result, current research trends are more focused on the development of non-toxic, environmentally friendly silicon-based materials to meet the modern electronics industry’s demand for high-temperature performance and flexibility.

Silicone rubber is an elastomeric polymer composed of an inorganic Si-O-Si main chain and organic side chains, combining excellent chemical stability, flexibility, processability, and non-toxicity [7,8,9]. It generally can only be used at temperatures up to 200 °C for extended periods [10]. In the field of flexible electronics [11,12], there are many high-temperature applications above 300 °C. During use, conductive silicones inevitably undergo aging and degradation, leading to changes in their electrical properties and physicochemical characteristics, which affect their service life and reliability. Therefore, there is an urgent need to improve the high-temperature resistance of silicone rubber materials. In research on enhancing the heat resistance of silicone rubber, traditional methods often rely on adding reinforcing fillers [13,14,15,16,17] to construct thermally stable networks, thus improving their thermal stability and mechanical properties. Gan et al. [18] introduced functionalized graphene oxide into the phenyl silicone rubber system, raising the initial decomposition temperature from 380 °C to 420 °C. Lou et al. [17] synthesized silicone rubber composites by adding a combination of glass fiber and glass powder fillers, achieving a char residue of 57.5% at 700 °C, which is significantly higher than the residue of pure silicone rubber. However, research on improving the high-temperature resistance of silicone rubber through molecular-level chemical modification [19,20] is relatively limited, leaving room for further exploration. The polysiloxane backbone structure features a highly flexible Si–O–Si framework, and its thermal properties are closely related to the chemical structure of the side groups. Common side groups include methyl, vinyl, and phenyl, and the introduction of different substituents can modulate key properties such as the thermal performance and mechanical strength of polysiloxane [21,22,23,24,25]. Among them, the introduction of phenyl substituents into the silicone rubber matrix significantly enhances the material’s thermal oxidative stability and thermal decomposition temperature compared to traditional silicone rubber [26,27] while also imparting higher mechanical strength to the material [28,29,30]. Wang et al. [16] investigated the effect of moderate to low phenyl content on the thermal stability of silicone rubber materials, finding that silicone rubbers containing 5% to 20% phenyl content achieved an initial decomposition temperature of up to 451 °C. Sheng et al. [31] studied the impact of phenyl groups on the thermal stability and mechanical properties of composite materials, with phenyl-containing composites displaying the highest initial decomposition temperature of 454 °C and excellent flexibility, while tensile strength, elongation at break, and hardness reached 3.10 MPa, 145%, and 56 A, respectively. These studies demonstrate that the incorporation of phenyl side groups into the silicone rubber molecular chain structure can effectively and significantly improve its high-temperature resistance.

Currently, high-temperature flexible conductive adhesive materials are generally based on organic silicon materials, with their conductivity enhanced by adding conductive fillers [32,33,34,35]. For example, Li et al. [36] investigated the conductivity of carbon nanotube/polydimethylsiloxane (PDMS) composites at various loadings. When the carbon nanotube content reached 0.2%, the conductivity of the composites increased sharply, forming a conductive network, with conductivity around 1 S/cm. Dou et al. [37] deposited silver nanoparticles onto carbon black and modified them with KH-590 to synthesize a branched grape-like structure filler (SMCB@Ag). The conductive silicone rubber obtained had a percolation threshold of 5.5 wt%, and when the content of SMCB@Ag increased to 10 wt%, the conductivity approached 10 S/cm. However, the 5% thermal weight loss temperature of the composite was only 350 °C, and the solid residue at 800 °C was only 31%. Yang et al. [38] developed a high-temperature-resistant composite by incorporating alumina–polyphenylene diamine–silver hybrids, which increased the initial decomposition temperature from 390 °C to 488 °C, with a solid residue of 62% at 800 °C. However, the composite’s conductivity was only 2.14 × 10^−12^ S/cm. Traditional heat-resistant or conductive fillers in silicone rubber often struggle to simultaneously improve both thermal stability and conductivity. In contrast, needle-like silver-coated glass fiber conductive fillers, with a high aspect ratio, facilitate the construction of a three-dimensional interconnected conductive network within the silicone rubber matrix. Their low percolation threshold characteristic allows continuous and efficient charge transport paths to be achieved with a lower filler content, thereby significantly enhancing the conductivity of the silicone rubber without sacrificing thermal stability.

Early studies on the heat resistance of silicone rubber mainly focused on introducing traditional inorganic fillers to enhance its thermal stability. However, research on enhancing the high-temperature properties of silicone rubber through chemical modification by introducing phenyl groups is relatively scarce. In this study, phenyl groups were introduced into the molecular structure of silicone rubber through chemical modification. A base polymer consisting of end-hydroxy silicone oil with a phenyl group content of 40% was used, and vinyltrimethoxysilane (VDMS) was used as a chain extender. Through a silanol condensation reaction, vinylmethylphenylpolysiloxane (VMPPS) with a crosslinkable structure was synthesized. This method enhances both the thermal stability and mechanical properties of the polymer while retaining an appropriate vinyl content, providing reactive sites for subsequent high-temperature vulcanization and crosslinking. Additionally, a green and efficient chemical plating process was employed to prepare silver-coated glass fibers (AGFs) with a high aspect ratio as a functional conductive filler. The AGF was then blended with VMPPS, followed by high-temperature press vulcanization to fabricate high-phenyl-content high-temperature flexible conductive silicone rubber materials. The effects of the synthesis parameters of VMPPS and the AGF filler ratio on the thermal stability and conductivity were systematically evaluated. The results indicated that the introduction of a high phenyl content significantly improved the thermal decomposition temperature and mechanical strength of the silicone rubber matrix, while the AGF filler constructed an efficient conductive network, achieving excellent conductivity at a low percolation threshold while maintaining the material’s flexibility. The high-phenyl-content high-temperature flexible conductive silicone rubber material demonstrates broad potential for application in extreme service environments, such as high-temperature flexible sensors, aerospace electronic seals, and combined heat and power (CHP) equipment.

## 2. Materials and Methods

### 2.1. Materials

Hydroxy-terminated phenyl silicone oil (Mn = 16,426, phenyl content 40%) was purchased from Zhejiang Xin’an Chemical Group Co., Ltd., Zhejiang, China. Methyl vinyl dimethoxy silane (C5H12O2Si, 98%), dibutyltin dilaurate (DBTDL, 95%), 3-hydroxytyramine hydrochloride (dopamine hydrochloride DA, 98%), tris(hydroxymethyl)aminomethane (tris, ultrapure), ammonia solution (28–30%), and anhydrous glucose (98%) were purchased from Shanghai McLean Biochemical Technology Co., Ltd., Shanghai, China. Silver nitrate (98%) was purchased from Aladdin Reagents Co., Ltd., Shanghai, China. Glass fibers (Toray Industries, Inc., Japan, d = 5–6 μm, 300 mesh, length 50–60 μm) were used. Commercial silicone rubber (model 120-6, Mn = 326,936, and phenyl content 20%) was purchased from Anhui Aiota Silicone Oil Co., Ltd., Anhui, China. Hydroxy silicone oil was obtained from Shandong Jielefu New Materials, Shandong, China. γ-Methacryloxypropyltrimethoxysilane (KH-570) was purchased from Dongguan Kangjin New Materials Technology Co., Ltd., Dongguan, China. The diethyl bisulfide vulcanizing agent (DHBP-50B) was obtained from Changsha Zhongyi Chemical Co., Ltd., Changsha, China.

### 2.2. Preparation of Vinyl Methyl Phenyl Polysiloxane

**Figure 1:**
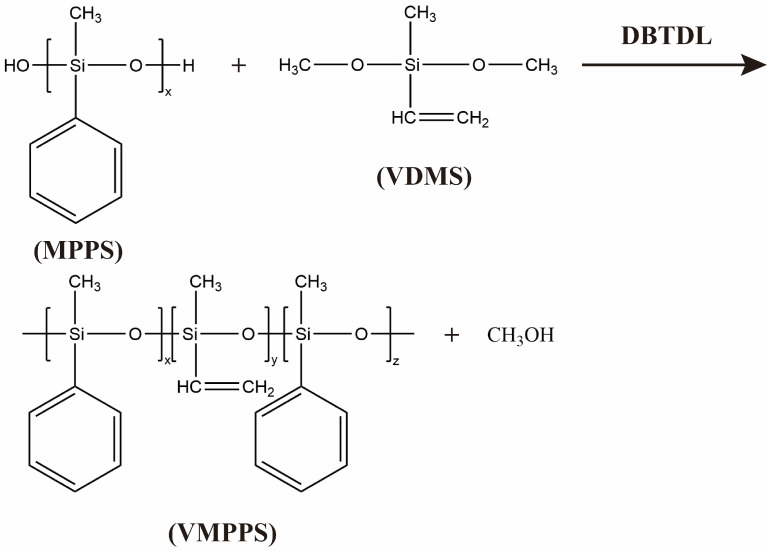
The synthesis route of VMPPS.

A specified amount of hydroxyl-terminated methyl phenyl polysiloxane (MPPS) and vinyl methyl dimethoxysilane (VDMS) is added to a 250 mL three-neck flask in a predefined feed ratio. The mixture is then equipped with a stirrer, condenser, and thermometer. After stirring for 5 min, dibutyltin dilaurate and a certain amount of deionized water are added. The reaction is carried out under reflux condensation at 100 °C. A vacuum distillation apparatus is installed, and the reaction proceeds under vacuum at a specified temperature for a set period, with a total reaction time of 40 h. After the reaction is completed, the catalyst is removed, and the product is transferred to a Petri dish. The dish is then placed in a vacuum drying oven and dried under vacuum for 6 h to obtain vinyl methyl phenyl polysiloxane (VMPPS). The synthesis process of VMPPS is shown in Figure 1.

### 2.3. Preparation of Chemically Plated Silver Glass Fibers

A total of 10 g of glass fibers (300 mesh, diameter approximately 5 μm) was sequentially dispersed in 50 mL of anhydrous ethanol and acetone, filtered, and dried at 50 °C to remove surface oil and processing agents. Subsequently, the fibers were treated in a 2 g/L dopamine (DA)–HCl solution (pH 8.5, 10 mM Tris-HCl) for 48 h, followed by ultrasonic treatment to remove aggregated polydopamine (PDA) particles. The silver plating precursor was used to convert Ag^+^ into silver oxide via hydroxide (OH^−^); then, ammonia (30%) was used to form the [Ag(NH_3_)_2_]^+^ complex. The PDA-coated fibers were dispersed in the silver precursor solution, and 50 mL of 2 mol/L glucose was added under stirring. After 20 min of reaction, the fibers were filtered, ultrasonicated, and washed three times with deionized water. Finally, the fibers were dried at 40 °C for 10 h to obtain silver-plated glass fibers (AGFs).

### 2.4. Preparation of Phenyl Silicone Rubber

**Figure 2:**
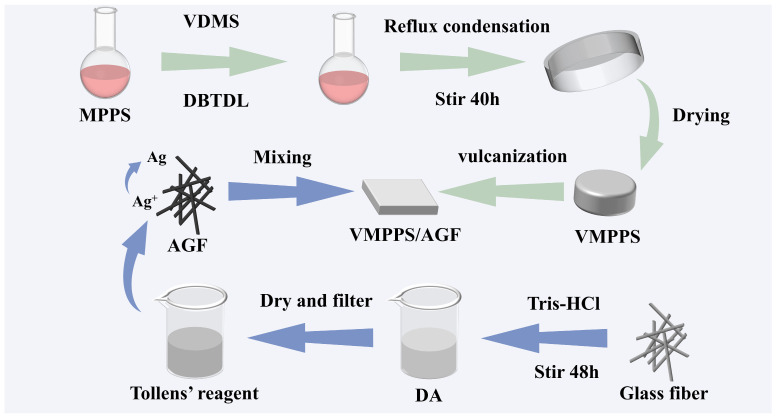
Schematic illustration of the fabrication process of the VMPPS silicone rubber.

The basic formulation consists of 100 parts of vinyl methyl phenyl polysiloxane (VMPPS), 25 parts of silver-plated glass fibers, 2 parts of hydroxyl silicone oil, and a curing agent (DHBP-50B). First, the VMPPS raw gum is added to a Banbury mixer and plastified to a translucent state to ensure proper material intake. Then, the needle-shaped silver-plated glass fiber conductive filler and a structure control agent are added, and the temperature is raised and maintained for a period to ensure uniform mixing. After cooling to room temperature, the curing agent is added, and the mixture is left at room temperature for 24 h. A two-stage vulcanization method is used: First, the compound is vulcanized and molded in a platen vulcanizer at 170 °C and 10 MPa, with the optimal cure time (tc90) and scorch time (tc10) determined using a rotorless vulcameter. Finally, the material undergoes second-stage vulcanization in an oven at 200 °C for 3 h. The overall preparation process of the VMPPS silicone rubber is shown in Figure 2.

### 2.5. Characterization

The samples were analyzed using Fourier transform infrared spectroscopy (FT-IR) on a Nicolet 6700 Fourier transform infrared spectrometer (Nicolet, Waltham, MA, USA, obtaining FT-IR spectra in the wavenumber range of 400–4000 cm^−1^. Proton nuclear magnetic resonance (^1^H-NMR) spectra were recorded on a Bruker Avance III HD spectrometer (Brucker Technology GmbH, Saarbrucken, Germany) at 400 MHz, using deuterated chloroform (CDCl_3_) as the solvent. The samples were dissolved in the THF solvent to a concentration of 2–4 mg/mL, allowed to stand until fully dissolved, and then filtered. Gel permeation chromatography (GPC) was performed using an Agilent 1260 Infinity II system (Agilent Technologies, Inc., Santa Clara, CA, USA) to determine the molecular weight and the molecular weight distribution. The GPC was run at a temperature of 40 °C with a flow rate of 1.0 mL/min. Conductivity was measured using a four-point probe resistivity tester (FT-341, RuiKe Instruments Co., Ltd., Ningbo, China). Scanning electron microscopy (SEM) was used to observe the morphology of the samples using a TESCAN MIRA LMS (TESCAN Group, Brno, Czech Republic). Thermal stability and high-temperature decomposition were analyzed using a thermogravimetric analyzer (NETZSCH STA449F3, Selby, Germany) by heating the sample from 30 °C to 800 °C at a rate of 10 °C/min in a nitrogen atmosphere. The dynamic mechanical properties of the vulcanized rubber were assessed using a DMA8000 dynamic mechanical analyzer (PerkinElmer, Waltham, MA, USA). Rectangular samples (100 mm × 5 mm × 2 mm) were subjected to temperature scanning at 10 Hz, in a temperature range of −130 °C to 100 °C, with a heating rate of 3 °C/min and a small strain of 0.1%. The strain sweep of uncured phenyl silicone rubber was performed using a Rubber Process Analyzer (RPA-8000, GOTECH, Taichung, Taiwan) at 60 °C and 1 Hz, with a strain range of 0.1–400%. The curing characteristics of AGF/phenyl silicone rubber materials were measured using an M-3000A rheometer (GOTECH, Taichung, Taiwan) at 170 °C for 30 min. The tensile strength and elongation at break of the vulcanized rubber were tested at 25 °C using a universal materials testing machine (MTSDW-20, Meitrs Testing Instruments Co., Ltd., Qingdao, China), with a tensile rate of 500 mm/min. Hardness was measured using a Shore A durometer (LX-A, Shanghai Gaozhi Precision Instruments Co., Ltd., Shanghai, China). The thermal aging tests involved placing the samples in an aging chamber at 25 °C for 24 h, 25 °C for 72 h, 100 °C for 24 h, and 100 °C for 72 h, followed by cooling to room temperature and allowing the samples to stand for 24 h before conductivity testing. The crosslinking density of the phenyl silicone rubber material in this study was determined using the equilibrium swelling method, with toluene as the solvent. The vinyl content was calculated using the standard solution titration method.

## 3. Results and Discussion

### 3.1. Preparation and Structural Characterization of Vinyl Methyl Phenyl Polysiloxane

To enhance the thermal stability and mechanical properties of silicone rubber materials under high-temperature environments, this study employs chemical modification by introducing rigid phenyl side groups into the silicone rubber system. This modification improves the thermal stability of the main chain and suppresses the thermal motion of the molecular chains. This study uses high-phenyl-content hydroxyl-terminated silicone oil (MPPS) as the base polymer and vinyl methyl dimethoxysilane (VDMS) as a chain extender to synthesize a crosslinkable phenyl polysiloxane (VMPPS) with high molecular weight and appropriate vinyl content through a silanol condensation reaction. The synthesis process introduces phenyl structural units while also providing vinyl reaction sites, which serve as effective crosslinking active sites for subsequent high-temperature vulcanization, thus providing a key intermediate for the preparation of high-performance heat-resistant phenyl silicone rubber. The feed ratio, vinyl content, molecular weight, and other related results are shown in Table 1. The base polymer, MPPS, exhibited an initial number-average molecular weight of 16,426. Upon the addition of VDMS and subsequent condensation chain extension, the molecular weight of the resulting VMPPS significantly increased. With the incremental addition of VDMS, the polymer chains of VMPPS progressively grew, leading to a gradual increase in Mn. When the feed ratio of MPPS to VDMS reached 100/12, the maximum molecular weight attained was 181,136, indicating a highly effective chain extension process. As the molecular weight increased, the physical state of the product transitioned from a low-viscosity fluid to a high-viscosity semi-fluid and eventually to an elastic solid at room temperature. This transformation is attributed to the enhanced entanglement density of the polysiloxane chains and stronger intermolecular interactions. These structural changes are beneficial for improving crosslinking efficiency and network stability during subsequent high-temperature vulcanization, thereby providing a robust molecular framework and favorable processability for the fabrication of high-temperature-resistant conductive silicone rubber materials. When the addition of VDMS is low, parts of the hydroxyl-terminated groups react with VDMS to form Si-O-Si bonds. However, due to the insufficient amount of VDMS, unreacted Si-OH groups remain in the system. A small number of siloxane molecules may undergo condensation reactions, leading to a slight increase in molecular weight. As the VDMS amount increases and its molar amount exceeds the hydroxyl-terminated groups, excessive VDMS hydrolyzes in the presence of water, generating Si-OH groups. These Si-OH groups further condense to form Si-O-Si network structures, significantly increasing the molecular weight. When the VDMS addition is too large, a large number of oligomers generated by hydrolysis cannot fully participate in condensation, leading to the formation of many small molecular by-products, ultimately resulting in a decrease in molecular weight. Additionally, the reaction temperature, reaction time, catalyst amount, and timely removal of by-products such as methanol all affect the extent of the condensation reaction and the final molecular weight of the product. When the addition of VDMS is controlled within the range of 4–16 wt%, the reaction proceeds sufficiently, and the resulting product exhibits solid-state elastomer characteristics at room temperature, with good processing stability. The vinyl content in the synthesized VMPPS increases with the amount of VDMS used. Initially, the increase is rapid, as the concentration of VDMS in the system rises with the increase in its usage, thus increasing the probability of a reaction with silanol groups, leading to a rapid rise in vinyl content. However, in the later stage, the increase rate slows down because the reaction probability does not increase indefinitely. Once it reaches a certain level, the vinyl content increase stabilizes.

**Figure 3:**
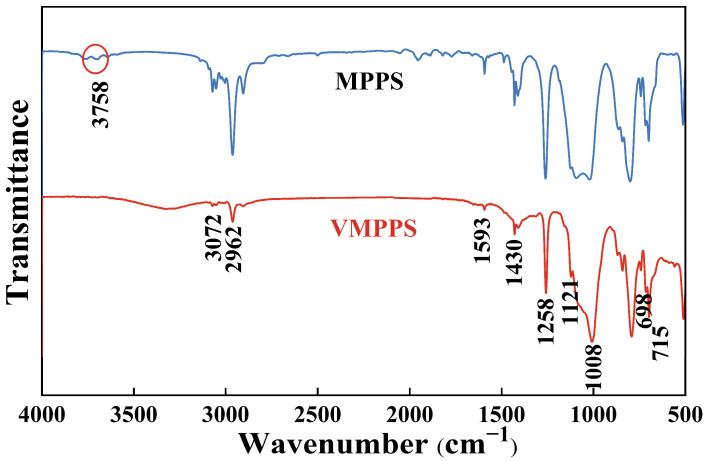
FT-IR curves of MPPS and VMPPS.

To verify the completion of the VMPPS synthesis process, Fourier transform infrared spectroscopy (FT-IR) and proton nuclear magnetic resonance (^1^H-NMR) analyses were performed on both the reactants and the VMPPS-M3 sample. The FT-IR spectra of MPPS and VMPPS are shown in Figure 3. As observed in Figure 3, the synthesized VMPPS shows a broad single peak in the range of 1000–1200 cm^−1^, corresponding to the characteristic absorption peak of the Si–O–Si structure. The absorption peaks at 1430 cm^−1^ and 1593 cm^−1^ correspond to the scissoring vibrations of the vinyl C=C bond bonded to the silicon atom and the conjugated C=C bond of the phenyl ring, respectively. The absorption peak at 3071 cm^−1^ corresponds to the stretching vibrations of C–H bonds in both the phenyl ring and the vinyl group, while the peak at 2969 cm^−1^ is primarily attributed to the stretching vibrations of C–H bonds in the Si–CH_3_ groups. No characteristic O–H stretching vibration peak for Si–OH groups was observed at 3758 cm^−1^ in the synthesized VMPPS, indicating that the condensation reaction between Si–OH groups was thorough, with almost no hydroxyl group residues.

**Figure 4:**
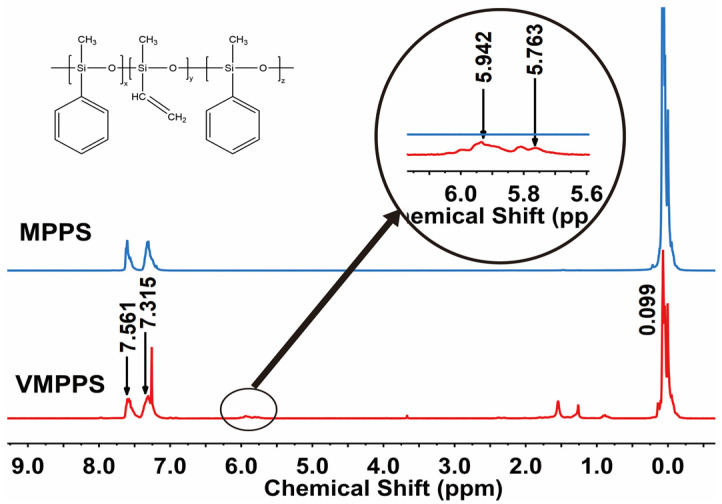
^1^H-NMR spectra of MPPS and VMPPS.

The proton nuclear magnetic resonance (^1^H-NMR) spectra of MPPS and VMPPS are shown in Figure 4. The synthesized VMPPS exhibits characteristic signals in the range of 0–0.3 ppm, corresponding to the hydrogen atoms directly bonded to the silicon atom in the methyl group (Si–CH_3_). The peaks at 5.763 ppm and 5.942 ppm correspond to the characteristic peaks of the methylene hydrogens (–CH_2_–) and the methine hydrogens (–CH–) of the vinyl group, respectively. The signal peaks between 7.0 and 7.8 ppm correspond to the hydrogens on the phenyl ring bonded to silicon (Si–Ph). Therefore, the infrared and ^1^H-NMR spectra of the synthesized product show excellent agreement with the characteristic absorption peaks of the functional groups, confirming that the synthesized product is VMPPS. The actual content of functional groups in the synthesized VMPPS was calculated from the integration of the characteristic absorption peaks in the ^1^H-NMR spectrum. Based on the integration areas of the functional group peaks in the ^1^H-NMR spectrum, the vinyl content was calculated, as shown in Table 1, which is in good agreement with the results obtained from the vinyl titration experiment.

### 3.2. Curing Characteristics and Crosslinking Density of Phenyl Silicone Rubber with Different Molecular Weights and Vinyl Contents

**Figure 5:**
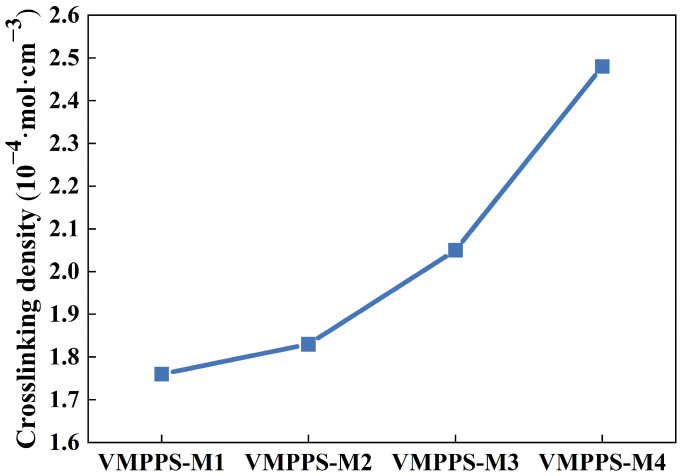
Crosslinking density of VMPPS/AGF silicone rubber.

The curing process of silicone rubber significantly affects the physical and mechanical properties of the material after molding. To explore the curing process and optimize curing temperature and time, vinyl methyl phenyl polysiloxane (VMPPS) with different molecular weights and vinyl contents, along with 25 parts of silver-coated glass fibers, 2 parts of hydroxyl siloxane, and 3 parts of the dicumyl peroxide curing agent, was subjected to curing in a rotorless rheometer at 170 °C to obtain the curing curves. The curing characteristics of VMPPS with different molecular weights and vinyl contents are shown in Table 2. The scorch time (t_c10_) for all four samples remained around 23 s, and the cure time generally ranged from 9 min. Furthermore, the difference in torque (MH-ML) can reflect the crosslinking density of the material [39]. It was observed that as the vinyl content increased, the torque difference gradually rose, indicating a corresponding increase in crosslinking density. This is due to the increased vinyl content and molecular weight, which enhances the crosslinking density, suggesting a higher level of crosslinking between AGF and the VMPPS matrix and stronger interactions at the filler–silicone rubber interface. As shown in Figure 5, the crosslinking density exhibited an increasing trend with the increase in vinyl content, which matched the variation in torque difference. This is because the peroxide curing agent in silicone rubber decomposes at high temperatures and pressure, generating free radicals that react with different molecular chains, leading to crosslinking. The higher the vinyl concentration in the system, the higher the crosslinking density. Within a certain range, appropriately increasing the crosslinking density facilitates the formation of a compact and stable three-dimensional network structure, thereby enhancing the mechanical strength and thermal stability of the material. However, when the vinyl content becomes excessively high, as observed in the VMPPS-M4 sample, over-crosslinking occurs in the system. This leads to a crosslinked structure that exceeds the optimal balance point for material performance, resulting in reduced chain segment flexibility and a more rigid, brittle network. Consequently, the material exhibits pronounced brittleness, with significantly decreased tensile strength and elongation at break.

### 3.3. The Processability of Phenyl Silicone Rubber with Different Molecular Weights and Vinyl Contents

**Figure 6:**
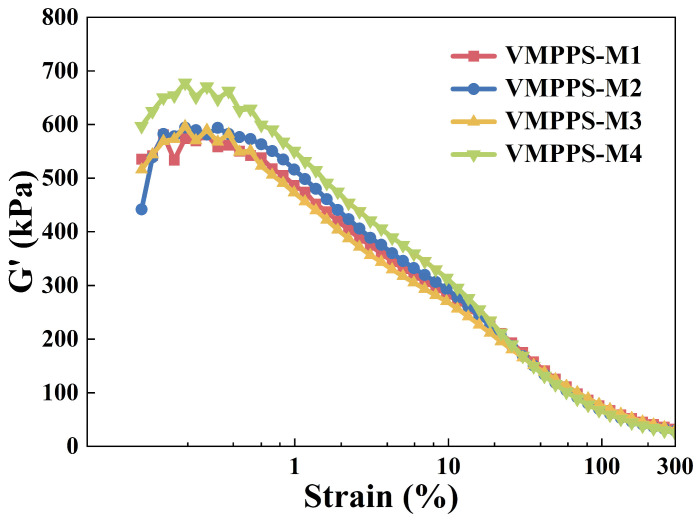
Strain amplitude dependence of uncured VMPPS/AGF silicone rubber.

To investigate the processing characteristics of silver-coated glass fiber (AGF) fillers in phenyl silicone rubber materials, vinyl methyl phenyl polysiloxane (VMPPS) was used as the matrix, with 25 parts of the AGF, 2 parts of hydroxyl silicone oil, and 3 parts of the DHBP-50B curing agent, and the mixture was high-temperature cured at 170 °C. Figure 6 illustrates the strain amplitude dependence of phenyl silicone rubber materials with different molecular weights and vinyl contents. As shown in Figure 6, at the same strain, VMPPS-M4 exhibits a higher G’ value, indicating an enhanced Payne effect. In the VMPPS system with excessively high vinyl content, the interaction at the filler–matrix interface is stronger, which negatively affects the dispersion of the filler in the matrix. In contrast, VMPPS-M1, VMPPS-M2, and VMPPS-M3 exhibit lower and comparable G’ values, with VMPPS-M3 having the lowest G’ value, suggesting that the AGF is better dispersed, and the material has good processing stability.

### 3.4. Mechanical Properties of VMPPS Silicone Rubber

**Figure 7:**
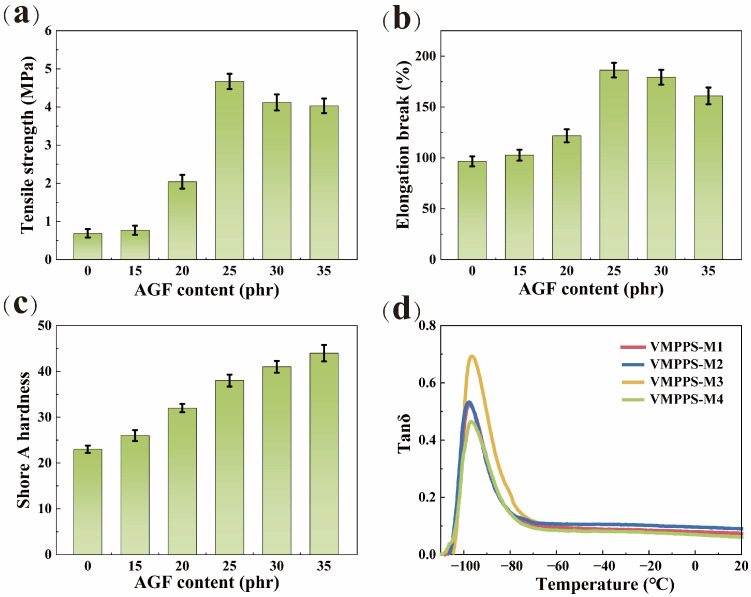
Histogram of (**a**) tensile strength, (**b**) elongation at break, and (**c**) Shore A hardness of VMPPS-M3 silicone rubber with different AGF contents; (**d**) DMA temperature sweep results of the VMPPS silicone rubbers.

The dispersion of the chemically plated silver glass fiber (AGF) in the silicone rubber matrix is reflected in its performance. To investigate the effect of different AGF contents on the mechanical strength of vulcanized phenyl silicone rubber, VMPPS-M3 with the highest molecular weight and appropriate vinyl content was used as the matrix. Three parts of a sulfur crosslinking agent and two parts of a structural control agent were added, followed by vulcanization at 170 °C. The detailed mechanical properties of the resulting phenyl silicone rubber are shown in Figure 7a–c. As the AGF content increases, the hardness of the phenyl silicone rubber gradually rises. When 15 to 25 parts of the AGF are added, both tensile strength and elongation at break show an increasing trend. However, when the AGF content exceeds 25 parts, the tensile strength and elongation at break begin to decrease. It can be observed that the mechanical properties of the phenyl silicone rubber are optimal with 25 parts of the AGF, with a tensile strength of 4.67 MPa, an elongation at break of 186.26%, and a hardness of 38 A. A further addition of the AGF leads to increased agglomeration of the AGF in the phenyl silicone rubber, resulting in poorer dispersion, stress concentration, and a decrease in tensile properties and elongation, ultimately compromising the material’s performance.

To further investigate the influence of the VMPPS matrix with different molecular weights and vinyl contents on the dynamic mechanical properties of vulcanized phenyl silicone rubber, 25 parts of the AGF were added as a filler, while other conditions remained unchanged. The temperature dependence of the phenyl silicone rubber materials is shown in Figure 7d. During the temperature scan tests, the maximum value of the loss factor was observed for all four compositions. This peak appeared during the glass transition process of the rubber composite, at which point the flowability of the rubber molecules rapidly increased, and the motion of the rubber molecules produced hysteretic losses [40]. With the increase in vinyl content, the tanδ value exhibits a trend of first increasing and then decreasing. The VMPPS-M3 sample shows the highest tanδ peak, while further increasing the vinyl content leads to a decline in tanδ. The moderate introduction of vinyl groups enhances the flexibility and free volume of the polysiloxane chains, facilitating the cooperative relaxation of more chain segments near the glass transition temperature (T_g_), thereby increasing the tanδ peak value. Meanwhile, the good dispersion of AGF (silver-coated glass fiber) conductive fillers within the matrix reduces filler agglomeration, allowing more silicone chains to move freely and participate in energy dissipation. However, excessive vinyl content, as in the VMPPS-M4 sample, leads to the over-crosslinking of the system, forming an overly dense three-dimensional network structure. This significantly restricts the mobility of polymer chains, increases the elastic modulus of the material, and reduces internal friction, resulting in a decreased tanδ peak. Moreover, a high crosslinking density may adversely affect the dispersion state of the fillers, causing the AGF to aggregate within the matrix and further deteriorate the filler–matrix interfacial compatibility, which suppresses the enhancement of tanδ. Therefore, the VMPPS-M3 sample achieves an optimal balance between vinyl content and filler dispersion, exhibiting superior energy dissipation capability and dynamic mechanical performance.

Different types of fillers exhibit a significant influence on the mechanical properties of silicone rubber materials. The incorporation of fumed silica as a reinforcing filler can markedly enhance the mechanical strength of silicone rubber due to its high specific surface area and favorable interfacial interactions, with tensile strength exceeding 10 MPa in some cases [41]. Additionally, it contributes to improved thermal stability. However, as an inherently insulating filler, fumed silica does not impart electrical conductivity to the silicone matrix, thereby limiting its applicability in electronic devices that require electrical functionality, such as flexible electronics and wearable sensors. In contrast, silicone rubber composites filled with 40 phr of BN exhibit a tensile strength of 3.10 MPa and an elongation at break of 145%. While the introduction of BN effectively enhances the thermal conductivity of the composite, its reinforcement effect on mechanical properties is relatively limited, and its insulating nature similarly prevents the material from achieving electrical conductivity [38]. A more balanced performance is observed in phenyl silicone rubber systems filled with CeO_2_ and graphene [42]. This combination not only improves thermal stability and offers a certain level of electrical conductivity but also yields desirable mechanical properties, with a tensile strength of 4.67 MPa and an elongation at break of 180%. Nevertheless, due to filler aggregation and limited interfacial compatibility with the polymer matrix, the overall electrical conductivity of the composite remains suboptimal, which restricts its use in high-performance conductive silicone rubber applications. In comparison, needle-like silver-coated glass fibers (AGFs) serve as an effective conductive filler capable of forming continuous conductive pathways within the silicone matrix. The AGF offers excellent electrical performance while maintaining good filler dispersion and strong interfacial adhesion, significantly enhancing the mechanical strength of the composite. Moreover, the AGF exhibits favorable thermal stability under high-temperature conditions, enabling the material to retain both electrical conductivity and heat resistance. Therefore, AGF-based multifunctional filler systems show great promise for the development of high-performance silicone rubber materials tailored for demanding applications such as high-temperature flexible electronics, sensors, and aerospace-grade thermal sealing components.

### 3.5. Conductivity of VMPPS Silicone Rubber

**Figure 8:**
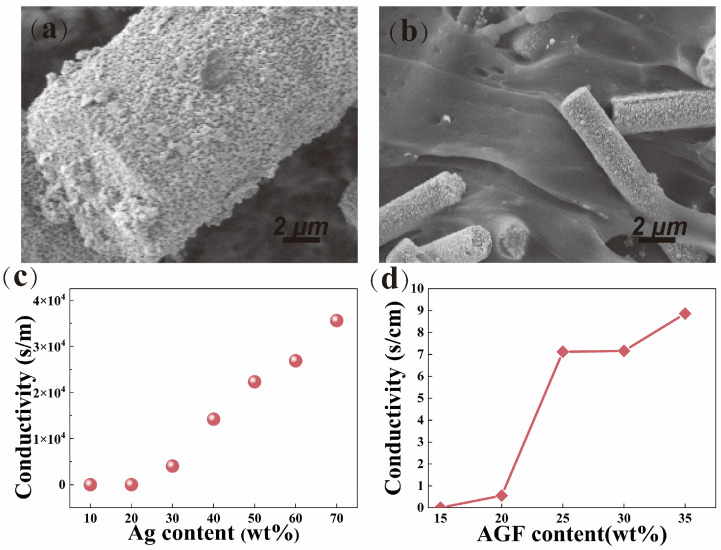
(**a**) SEM images of the AGF; (**b**) SEM images of VMPPS/AGF; (**c**) conductivity of the AGF as a function of the mass fraction of Ag; (**d**) Changes in conductivity of AGF-VMPPS at different mass fraction contents of the AGF.

To overcome the adverse effects of traditional heat-resistant fillers on the electrical conductivity of silicone rubber while enhancing thermal stability, this study employs an environmentally friendly and efficient chemical plating process to prepare high-aspect-ratio needle-like silver-plated glass fibers (AGFs) as conductive fillers. These fillers not only effectively reduce the overall material cost but also enable the formation of an efficient and stable conductive network within silicone rubber. When incorporated into high-phenyl-content polysiloxane (VMPPS), it results in a high-temperature conductive phenyl silicone rubber material with excellent electrical conductivity and thermal stability, maintaining good electrical performance in high-temperature environments. In the preparation of the chemically silver-plated glass fibers, AgNO_3_ was used as the silver precursor, glucose as the reducing agent, and ammonium hydroxide as the complexing agent. The complexation with AgNO_3_ forms a silver–ammonia solution, thereby controlling the self-reduction of AgNO_3_. The SEM image of the glass fibers is shown in Figure 8a, where the surface of the glass fibers is observed to be smooth and clean. The electrical conductivity of the silver-plated glass fibers with different silver content mass fractions is shown in Figure 8c. When the Ag content is below 20%, the conductivity of the silver-plated glass fibers is relatively low (5–10 S/m). As the Ag content increases, the conductivity gradually rises; this is attributed to the extremely high electrical conductivity of metallic silver [43,44]. When the Ag content reaches 40%, the conductivity rapidly increases to 1.42 × 10^4^ S/m. This is because, at low Ag content, the PDA layer’s adsorption capacity for Ag^+^ is limited, leading to significant defects between silver particles, which hinders the effective formation of conductive pathways. However, as the silver content increases, the polydopamine layer can fully adsorb Ag^+^, forming a silver layer with excellent conductivity and providing an efficient conductive pathway.

**Figure 9:**
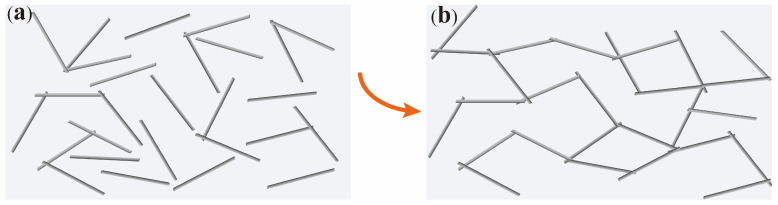
(**a**) Overlapping state of the AGF at low loadings (no conductive pathway formed); (**b**) overlapping state of the conductive AGF at a percolation threshold (continuous conductive network formed).

Using VMPPS-M3 with the highest molecular weight and suitable vinyl content as the matrix, three parts of DBPH, two parts of hydroxyl silicone oil, and varying amounts of the AGF were added and vulcanized at high temperatures. Figure 8d illustrates the relationship between the AGF content and the electrical conductivity of the vulcanized phenyl silicone rubber. It can be observed that at lower AGF contents, effective contact within the silicone rubber matrix was not achieved, and a good conductive network was not formed. Therefore, the electrical conductivity of the VMPPS/silver-plated glass fiber phenyl silicone rubber did not show significant changes. When the AGF content was 15 parts, the conductivity was only 2.53 × 10^−3^ S/cm. As the AGF content gradually increased, the conductivity of the phenyl silicone rubber reached 7.12 S/cm at 25 parts, while at 20 parts, the conductivity was only 0.556 S/cm. When the AGF content was 30 and 35 parts, the conductivities were 7.16 S/cm and 8.86 S/cm, respectively. However, with the further increase in AGF content, the rate of conductivity enhancement gradually levels off, indicating that the percolation threshold of the VMPPS/silver-plated glass fiber phenyl silicone rubber material was reached at 25 parts. Sufficient contact between fibers allowed for the formation of an excellent conductive network, which greatly enhanced conductivity. The SEM image in Figure 8b further confirms this observation. VMPPS/AGF phenyl silicone rubber with different AGF contents and commercial rubber 120-6 with 25 parts of the AGF were placed in a constant temperature oven and subjected to heat aging at 25 °C for 24 h, 25 °C for 72 h, 100 °C for 24 h, and 100 °C for 72 h, followed by electrical conductivity tests. The results are shown in Table 3. For the same 25 parts of the AGF, after heat aging at 25 °C and 100 °C, the electrical conductivity decay of the VMPPS-M3 phenyl silicone rubber was much lower than that of the commercial rubber, demonstrating excellent conductive stability. Figure 9 illustrates the schematic evolution of the conductive network structure with the gradual addition of AGF fillers, clearly revealing the formation process of conductive pathways from isolated distribution to continuous percolation.

### 3.6. Heat Resistance of VMPPS Silicone Rubber

**Figure 10:**
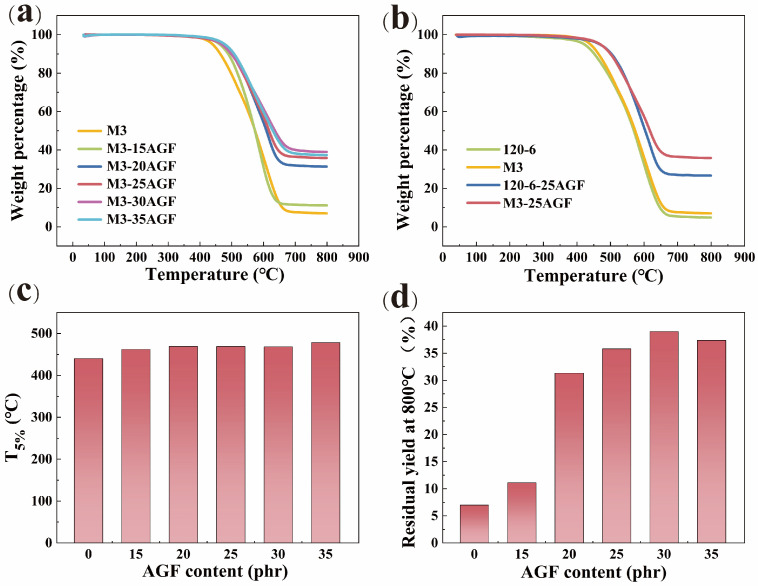
Thermal stability of materials: (**a**) TGA curves of VMPPS-M3/AGF; (**b**) TGA curves of VMPPS-M3/AGF and 120-6/AGF; (**c**) temperature at which 5% mass loss occurs (T_5%_); (**d**) residual yield at 800 °C.

Flexible electronic devices often need to withstand thermal stresses of up to 200 °C or even higher during long-term service. To meet the stringent requirements for material properties in high-temperature environments, research on the heat resistance of silicone rubber is essential. Using VMPPS-M3 as the base rubber, different amounts of AGF fillers, three parts of dicumyl peroxide, and two parts of hydroxyl silicone oil were added to process the silicone rubber, which was then characterized for its thermal properties after vulcanization using TGA. Figure 10a shows the TGA results for various amounts of AGF added. The heat resistance of the phenyl silicone rubber containing AGF fillers showed significant improvements compared to pure VMPPS phenyl silicone rubber. When 35 parts of the AGF were added, the T_5%_ was as high as 478 °C, with a mass residue of 37.36% at 800 °C, both of which were much higher than the VMPPS phenyl silicone rubber without AGF fillers. As shown in Figure 10b, M3 and 120-6 represent the components without fillers. The T_5%_ of the silicone rubber made from commercial 120-3 without the AGF was 421 °C, with a mass residue of 4.81% at 800 °C. After adding 25 parts of the AGF, the T_5%_ of the vulcanized silicone rubber reached 460 °C, with a mass residue of 26.65% at 800 °C. On the other hand, for the self-made VMPPS-M3, the T_5%_ of the vulcanized silicone rubber without the AGF was 440 °C, with a mass residue of 6.99% at 800 °C. After adding 25 parts of the AGF, the T_5%_ reached 469 °C, and the mass residue at 800 °C was 35.81%, which was superior to the commercial 120-6 and far higher than the VMPPS without AGF fillers. Therefore, the addition of AGF fillers can enhance the heat resistance of VMPPS phenyl silicone rubber materials, and the self-made VMPPS demonstrates better thermal stability at high temperatures compared to commonly used commercial rubbers.

## 4. Conclusions

In this study, a high-performance silicone rubber material was developed based on high-phenyl-content α and ω-dihydroxy-terminated polysiloxane through chemical modification via the introduction of vinyl functional groups. The modified silicone matrix was further reinforced with high-aspect-ratio needle-like silver-plated glass fibers (AGFs) as conductive fillers. This material system exhibits outstanding comprehensive properties, including excellent thermal resistance (T_5_% up to 478 °C), high electrical conductivity (7.12 S/cm), and favorable mechanical strength (tensile strength of 4.67 MPa), while maintaining stable electrical performance after long-term thermal aging at elevated temperatures. The synergistic enhancement strategy through molecular structure tailoring and the incorporation of anisotropic conductive fillers effectively addresses the long-standing issues of conductivity degradation and embrittlement in conventional conductive silicone rubbers under high-temperature environments. It achieves a balanced optimization and multifunctional integration of thermal stability, electrical conductivity, and mechanical integrity. This research not only provides a promising structural design and processing strategy for the development of high-performance silicone rubber materials capable of long-term stability under extreme thermal and mechanical stresses but also lays the theoretical foundation and practical pathway for the fabrication of high-performance flexible electronic devices. The VMPPS/AGF composite system demonstrates strong application potential in commercial sectors such as flexible electrodes, temperature sensors, and high-temperature-resistant aerospace sealing components.

## Figures and Tables

**Table 1:** Synthesis formulations of VMPPS with different vinyl contents and molecular weights.

Samples	Precursors	Product			
	MPPS/VDMS	Vinyl Content (%)	Mn (g/mol)	Đ	State at Room Temperature
VMPPS-M0	100/1	0.01	38433	3.41	Viscous liquid
VMPPS-M1	100/4	0.11	131591	2.49	Solid
VMPPS-M2	100/8	0.15	147178	2.58	Solid
VMPPS-M3	100/12	0.17	181136	2.43	Solid
VMPPS-M4	100/16	0.23	122031	2.88	Solid
VMPPS-M5	100/20	0.25	45827	2.83	Viscous liquid

**Table 2:** The curing characteristics of VMPPS/AGF silicone rubber.

Samples	t_c10_/s	t_c90_/min	M_L_ (dNm)	M_H_ (dNm)	M_H_-M_L_ (dNm)
VMPPS-M1	22	8.58	5.89	17.49	11.6
VMPPS-M2	24	8.75	5.95	18.68	12.73
VMPPS-M3	23	9.26	5.26	21.43	16.17
VMPPS-M4	22	5.67	6.45	35.61	29.16

**Table 3:** Conductivity loss rate of VMPPS with different AGF contents.

Conductivity Loss Rate/%	120-6-25AGF	M3-25AGF	M3-30AGF	M3-35AGF
25 °C for 24 h	1.82	1.24	1.12	1.35
25 °C for 72 h	6.63	5.22	5.43	5.36
100 °C for 24 h	18.12	14.65	15.86	15.21
100 °C for 72 h	36.58	30.92	32.16	34.46

## Data Availability

Data are contained within the article.
